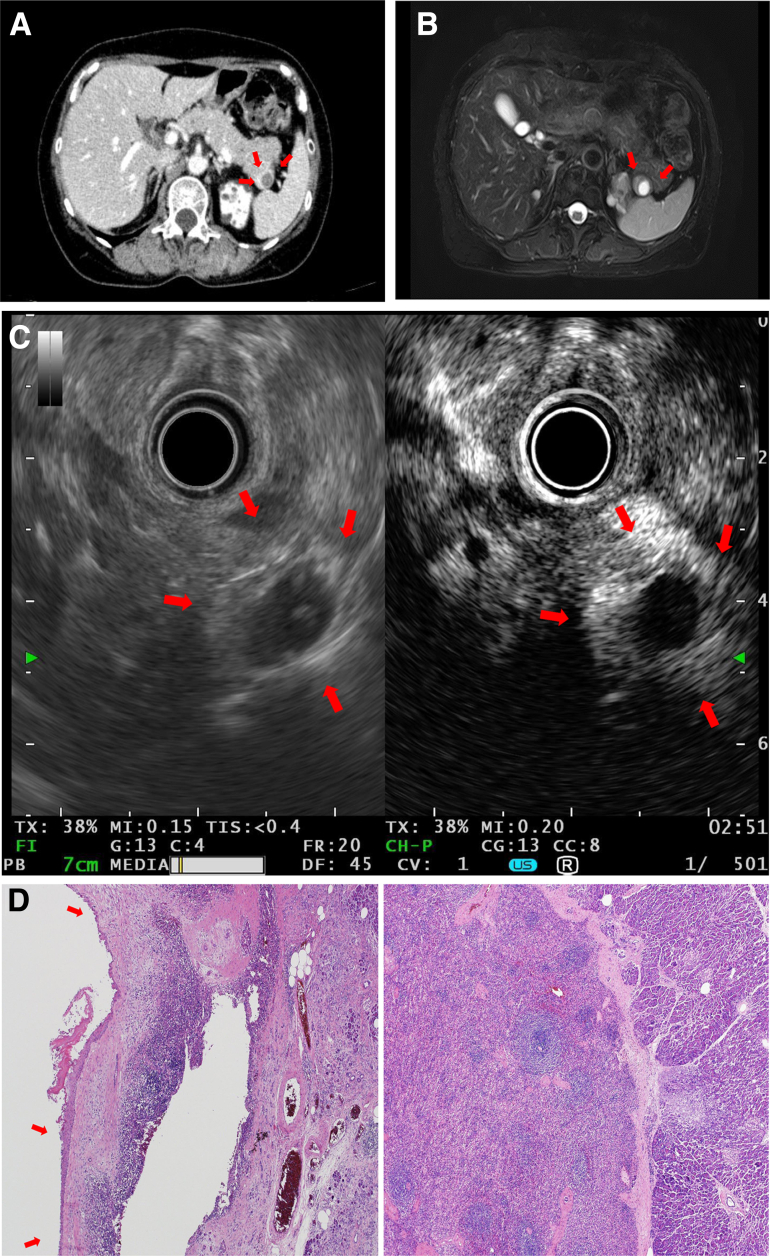# Epidermal Cyst in an Intrapancreatic Accessory Spleen

**DOI:** 10.1016/j.gastha.2022.09.007

**Published:** 2022-09-23

**Authors:** Shingo Ogiwara, Yusuke Nomoto, Taro Osada

**Affiliations:** Department of Gastroenterology, Juntendo University Urayasu Hospital, Chiba, Japan

A 59-year-old asymptomatic woman with no disease history presented to our institution for investigation of a pancreatic tail mass. Hematological parameters were within normal ranges, including serum carcinoembryonic antigen levels and carboxyhydrate antigen 19-9. Computed tomography revealed a strongly enhanced solid peripheral mass with an intracystic component in the pancreatic tail (Figure A, arrow). Magnetic resonance imaging revealed a cystic lesion on the T2 image surrounded by a solid component with density and intensity levels similar to those in the spleen parenchyma (Figure B, arrow). Endoscopic ultrasonography showed a solid hyperechoic 20-mm mass and an intracystic hypoechoic component showing stronger enhancement by Sonazoid (MSD Inc) than the pancreatic parenchyma (Figure C, arrow). Laparoscopic distal pancreatectomy was used for diagnosis. Pathological examination revealed a white and red sinus of the ectopic spleen in the pancreatic tail, with a unilocular cyst lined with stratified squamous epithelium, indicating an epidermal cyst in the intrapancreatic accessory spleen (Figure D, arrows).

Most epidermal cysts in the intrapancreatic accessory spleen cases are found incidentally, typically as single monolobular cystic lesions in the pancreatic tail with a thickened cystic wall or solid components identical in density to the spleen on imaging examinations, making a definite preoperative diagnosis extremely difficult, resulting in unnecessary surgical intervention.

**Figure:**